# Remnant salmon life history diversity rediscovered in a highly compressed habitat

**DOI:** 10.1111/eva.13741

**Published:** 2024-07-02

**Authors:** Sara A. Hugentobler, Anna M. Sturrock, Malte Willmes, Tasha Q. Thompson, Rachel C. Johnson, Flora Cordoleani, Natalie J. Stauffer‐Olsen, George Whitman, Mariah H. Meek

**Affiliations:** ^1^ Department of Integrative Biology Michigan State University East Lansing Michigan USA; ^2^ Center for Watershed Sciences, UC Davis Davis California USA; ^3^ School of Life Sciences University of Essex Colchester UK; ^4^ Institute of Marine Sciences University of California Santa Cruz California USA; ^5^ Norwegian Institute for Nature Research Trondheim Norway; ^6^ Wild Salmon Center Portland Oregon USA; ^7^ Southwest Fisheries Science Center National Marine Fisheries Service Santa Cruz California USA; ^8^ Trout Unlimited Emeryville California USA

**Keywords:** acoustic tagging, conservation genetics, GREB1L, life history diversity

## Abstract

Chinook salmon (*Oncorhynchus tshawytscha*) display remarkable life history diversity, underpinning their ability to adapt to environmental change. Maintaining life history diversity is vital to the resilience and stability of Chinook salmon metapopulations, particularly under changing climates. However, the conditions that promote life history diversity are rapidly disappearing, as anthropogenic forces promote homogenization of habitats and genetic lineages. In this study, we use the highly modified Yuba River in California to understand if distinct genetic lineages and life histories still exist, despite reductions in spawning habitat and hatchery practices that have promoted introgression. There is currently a concerted effort to protect federally listed Central Valley spring‐run Chinook salmon populations, given that few wild populations still exist. Despite this, we lack a comprehensive understanding of the genetic and life history diversity of Chinook salmon present in the Yuba River. To understand this diversity, we collected migration timing data and GREB1L genotypes from hook‐and‐line, acoustic tagging, and carcass surveys of Chinook salmon in the Yuba River between 2009 and 2011. Variation in the GREB1L region of the genome is tightly linked with run timing in Chinook salmon throughout their range, but the relationship between this variation and entry on spawning grounds is little explored in California's Central Valley. We found that the date Chinook salmon crossed the lowest barrier to Yuba River spawning habitat (Daguerre Point Dam) was tightly correlated with their GREB1L genotype. Importantly, our study confirms that ESA‐listed spring‐run Chinook salmon are spawning in the Yuba River, promoting a portfolio of life history and genetic diversity, despite the highly compressed habitat. This work highlights the need to identify and protect this life history diversity, especially in heavily impacted systems, to maintain healthy Chinook salmon metapopulations. Without protection, we run the risk of losing the last vestiges of important genetic variation.

## 1 INTRODUCTION

Life history diversity is critical for species to respond to environmental variability (Beechie et al., 2006; Moore et al., 2014). This diversity often includes differences in morphology, size, and age at maturity and is often influenced both by environmental and genetic factors (Healey, 1991; Thibaut & Connolly, 2013). In particular, genetic diversity is important because it often harbors the adaptive potential for populations to respond to future or changing conditions (Brooks et al., 2006; Chapin et al., 2000). Additionally, genetic diversity within a species or population can result in the expression of diverse life history strategies that spread survival risk across time and space, stabilizing populations and ecosystem services. This phenomenon is referred to as biocomplexity (Hilborn et al., 2003) and can help buffer the effects of natural and anthropogenic change (Narum et al., 2018). Unfortunately, biocomplexity, and in turn genetic diversity, is being lost at alarming rates due to anthropogenic change, particularly in freshwater ecosystems (Allendorf et al., 2014; Des Roches et al., 2021; Heino et al., 2009; Sih et al., 2000). To protect biocomplexity and promote life history diversity, it is vital to identify, monitor, and protect unique phenotypic and genetic traits present within and among populations.

In general, salmonids in the United States have been losing biocomplexity over the last century due to anthropogenic stressors (Dittman & Quinn, 1996; Finney et al., 2002; Malick & Cox, 2016). For example, Chinook salmon (*Oncorhynchus tshawytscha*) have faced declines in excess of 99% of their original population sizes in their native range due to overfishing, damming, mining, and climate change (Mahnken et al., 1998; National Marine Fisheries Service, 2014). This is particularly troubling because Chinook salmon are a keystone species of high cultural, economic, and ecological value (Bottom et al., 2009; Colombi, 2012; Layman et al., 2006). With large population losses, many Chinook salmon populations have also experienced a marked reduction in genetic diversity (Johnson et al., 2018; Thompson et al., 2019; Weeder et al., 2005). These significant losses in genetic diversity have had negative consequences in terms of reductions in phenotypic diversity and adaptive capacity (Carlson & Satterthwaite, 2011; Griffiths et al., 2014). Thus, it is vital that we identify and protect the remaining biocomplexity found in Chinook salmon populations to promote population persistence and resilience in an anthropogenically influenced system.

The California Central Valley (CCV) is the southernmost portion of the native Chinook salmon range, and populations are greatly imperiled due to the negative impact of anthropogenic stressors such as dams, historic mining operations, and extensive urbanization (Herbold et al., 2018; Moyle et al., 2017). Due to its southern location, Chinook salmon populations in the CCV are also highly vulnerable to climate change (Crozier et al., 2019). Despite these threats, the Sacramento River is the only part of the entire species' range that contains four distinct spawning life history timings, while all other systems have only two distinct run timings. This makes the Chinook salmon in the CCV a uniquely diverse population complex (Williams, 2006). These life history phenotypes are referred to as “run‐types” and are named after the season by which adults migrate upriver to spawn (fall, late fall, spring, and winter). Historical temporal and spatial separation has resulted in limited gene flow among CCV run‐types within the same river system, leading to these populations becoming genetically distinct (Meek et al., 2020). This genetic variation provides the adaptive capacity necessary to result in phenotypically diverse populations. This biocomplexity in run‐types is essential in maintaining Chinook salmon stock abundance across years, facilitating a “portfolio effect” that allows the species to withstand environmental heterogeneity and perturbations (Schindler et al., 2010). Although we know much about the biology of Chinook salmon, much is still unknown about the heritability or genetic basis of life history traits in Central Valley populations (Cordoleani et al., 2020).

Spring‐run Chinook salmon were once the most abundant run in the CCV, existing in the hundreds of thousands prior to the construction of impassable dams, extensive levees that converted floodplain and marsh habitat to agricultural land, and overfishing (Lindley et al., 2004; Yoshiyama et al., 1998). Spring‐run fish display a unique spawning strategy of migrating into the system early when water temperatures are low from high spring flows and oversummering in cool headwaters before spawning in the fall (Quinn et al., 2016). Unfortunately, dam construction in the CCV, which began in the early 1900s, cut off access to historical spring‐run Chinook salmon spawning habitat for most populations throughout the CCV. This forced spring‐run to face the double threat of both having to oversummer in much warmer downstream waters while also spawning in the same habitat as fall‐run Chinook salmon, which enter the system after the heat of the summer and spawn immediately in downstream reaches (Healey, 1991). Consequently, spring‐run numbers have decreased precipitously, with most populations going entirely extinct in the CCV (Williams, 2006; Yoshiyama et al., 1998). As a result, they are now listed as threatened under the Endangered Species Act (National Marine Fisheries Service, 2014).

The Yuba River, a tributary of the Feather River within the Sacramento River watershed, once supported an independent spring‐run population, but like much of the rest of the CCV, due to extensive damming, historic spring‐run spawning grounds are no longer accessible, making it an excellent system for identifying and understanding if and how various life history forms co‐exist in a heavily impacted system (James, 2005). The Yuba River Chinook salmon population is not currently considered two genetically distinct populations, despite the presence of early and late returning migrating adults (National Marine Fisheries Service, 2014). A key unknown is the extent of life history and genetic variation within the system. It has also been assumed to be largely influenced by strays from the nearby Feather River Hatchery, where there has been mixing of fall and spring‐run migration phentoypes in the past (Lindley et al., 2004). It is unknown if there is an independently spawning, genetically distinct spring‐run population in the Yuba River. If a genetically distinct spring‐run population exists in the Yuba River, it will be critical to manage this watershed appropriately to protect the ESA‐listed population and, in turn, promote the overall spring‐run genetic portfolio.

In recent years, notable progress has been made toward understanding the genetic underpinnings of run timing diversity in Chinook salmon. Research in other systems has shown that variation in return timing of fall and spring‐run Chinook salmon is tightly correlated with variation in the GREB1L to ROCK1 region of the genome located on Chromosome 28, hence referred to in this paper as GREB1L (Prince et al., 2017; Thompson et al., 2019). Chinook salmon homozygous for the early returning allele exhibit an early run timing distribution in the spring, while individuals homozygous for the late returning allele exhibit a later distribution in the fall. Heterozygotes in other systems exhibit an intermediate return timing that overlaps to some extent with homozygotes of both alleles (Prince et al., 2017; Thompson et al., 2019). Although this correlation has been well studied and documented in other river systems (such as the Rogue River, Oregon, and Klamath River, California) using well‐phenotyped samples from migrating adults, studies in the CCV to date have relied on phenotypic proxies for run timing, such as carcass collection date or entry time into a hatchery (Thompson et al., 2020). While previous work was sufficient to demonstrate the strong correlation of the GREB1L region with run timing in the CCV, the correlation between these proxies and freshwater entry weakens as the fish move further upstream and the migration season continues (Waples et al., 2022). We endeavored to meet these challenges by obtaining information from live individuals in the midst of their migration, providing more precise information about the timing distributions of each genotype. In this study, we sought to both identify how many migration phenotypes are present in the Yuba River and to explore the relationship between GREB1L genotypes and the return time of Chinook salmon in the CCV. To achieve this, we genotyped individuals across three different points in their migration—as they first entered the Yuba watershed, as they crossed barriers to higher spawning grounds, and after spawning. Using three different points of reference, we further elucidated the relationship between GREB1L genotypes and migration. Understanding this in the highly impacted Yuba River system is invaluable for not only the management of the Yuba River, given the rarity of the spring‐run, but is also important for understanding how life history diversity is maintained in highly impacted systems. This knowledge will help researchers and managers determine how to identify, monitor, and protect this life history diversity to promote salmonid recovery.

## 2 METHODS

### 2.1 Study site

**FIGURE 1:**
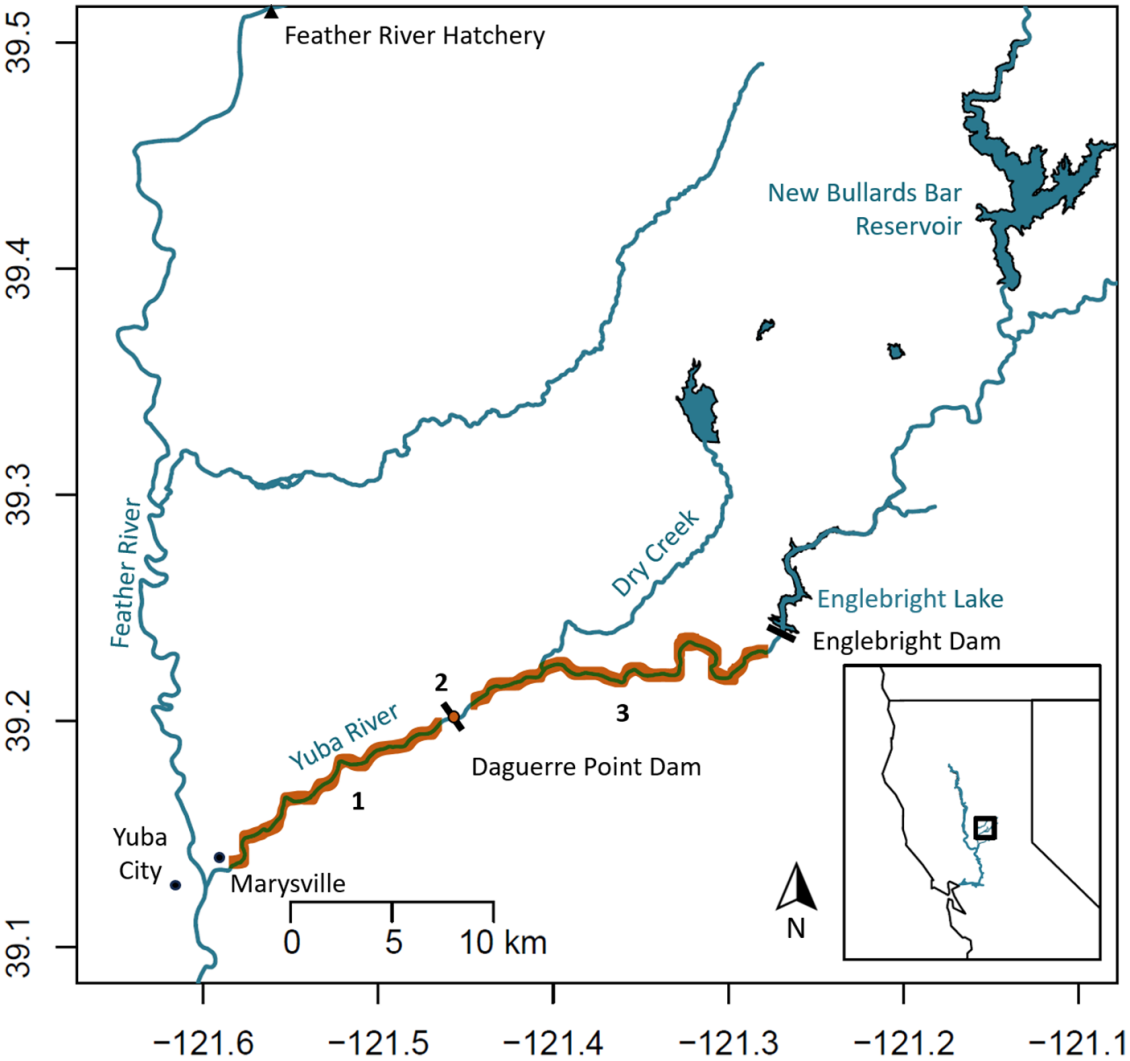
Map of the Yuba River system, a tributary of the Feather River. Black bars indicate dams. Orange highlighted areas indicate sampling locations: (1) hook‐and‐line survey sampling location, (2) acoustic tagging sampling area, and (3) carcass sampling area.

The Yuba River is a tributary of the Feather River, which flows into the Sacramento River. The Yuba has three main tributaries, the north, middle, and south forks, which were once historic Chinook salmon‐spawning habitat but are now inaccessible due to dams on the river. The Yuba River has two main dams that serve as barriers to Chinook salmon migration: the Daguerre Point Dam (DPD), which is located at river mile 11 and passable by salmon via two fish ladders on either side, and the Englebright Dam, which is located at river mile 24 and impassable by salmon (Figure 1). In addition to these complications, upstream from the Yuba River confluence, there is a large hatchery located on the Feather River that produces both spring and fall‐run fish that are known to stray into the Yuba River during spawning migrations (Dean & Lindley, 2023). A key management objective in this system is the Yuba River Accord, which is an agreement between all agencies in the Lower Yuba River Management Team (RMT) to manage for improved salmon and steelhead habitat. Within the Yuba River Accord Fisheries Agreement, it is a stated purpose to evaluate the presence and viability of spring‐run Chinook salmon in the lower Yuba River (Yuba County Water Agency, 2007).

### 2.2 Sample collection

Samples were collected through three main sampling efforts: a hook‐and‐line survey, an acoustic telemetry project, and a carcass survey, conducted by the RMT between the years 2009 and 2011 as part of their annual surveys to characterize Chinook salmon migration up the Yuba River to the spawning reaches (Table 1). For the hook‐and‐line survey and acoustic telemetry effort, genetic samples were collected from all adult fish caught via hook‐and‐line sampling, targeting fish in the lowermost reaches from the confluence of the Yuba and Feather Rivers to DPD from May to October, 6 days a week during the years 2010–2011 (Sampling Area 1, Figure 1). Fin clips were collected from all captured fish (*N* = 122), but only fish that were determined to be in “good condition” (showing no signs of disease or injury) were selected to be acoustically tagged as part of the acoustic tagging survey effort (*N* = 42, we refer to these as the “acoustic tagging samples” and those that were just fin clipped but not tagged as “hook‐and‐line survey samples”). The acoustic tagging samples were tagged with VEMCO V13‐1 L acoustic transmitters via esophageal/gastric insertion and were detected via two ultrasonic receivers located in the north and south sides of the top of the fish ladder to detect fish successfully passing DPD from both sides (Sampling Area 2; PSMFC, 2011; VEMCO, 2010). The most upstream area was sampled via carcass surveys that occurred upstream of the DPD on a weekly basis during the years 2009–2010 (Sampling Area 3, Figure 1), starting 10–15 days after the first spawning redds were detected each year. Only fresh carcasses (possessing at least one clear eye and gills that are red or pink) were sampled to avoid sampling fish that had degraded DNA and had already been in the system for a long period of time. In 2009 and 2010, tissue samples were taken from carcasses throughout the river reach between the DPD and Englebright Dam (Sampling Area 3). To mitigate the possibility of hatchery fish from the Feather River Hatchery (or other hatcheries) being included in our analysis, fish with their adipose fin clipped were excluded. This reduces, but does not totally exclude all hatchery‐origin fish, since only 25% of all fall‐run hatchery‐origin fish have their adipose fins clipped. All tissue samples, regardless of survey method, were dried and placed into individual envelopes, and then sent to the Meek genetics lab at Michigan State University for processing.

**TABLE 1:** Samples collected and genotyped.

Sample year	Survey type	Sampled *N*	Genotyped *N*
2009	Hook‐and‐line Survey	0	NA
	Acoustic Tagging	0	NA
	Carcass Survey	42	37
	Total	42	37
2010	Hook‐and‐line Survey	95	92
	Acoustic Tagging	18	18
	Carcass Survey	38	35
	Total	133	127
2011	Hook‐and‐line Survey	44	30
	Acoustic Tagging	26	24
	Carcass Survey	0	NA
	Total	44	30
Total		219	194

*Note*: Numbers are presented by year and survey type. Note that acoustic tagging individuals were first surveyed in the hook‐and‐line survey and then again when they passed DPD, and as such are a portion of the hook‐and‐line survey individuals. Salmon with an adipose fin clip were excluded.

### 2.3 Run‐type assignment

We first assigned individuals to phenotypic run‐timing by the date of their detection in the system. The Yuba River RMT uses two “differentiation days” to classify individuals into either the spring early, spring late, or fall‐run timing categories. If an individual fish passes DPD prior to July 15th, they are considered spring early run migrants, while after that but prior to October 1st, they are considered spring late run migrants. All fish after October 1st are considered fall‐run migrants (Poxon & Bratovich, 2020). We used these same metrics to classify individuals according to their phenotypic run‐timing and compare them with their GREB1L genotypes. Although not typically used for fish below the DPD, to compare results between these samples and those when they passed the dam, we used this same method of classification for fish surveyed below the DPD.

**FIGURE 2:**
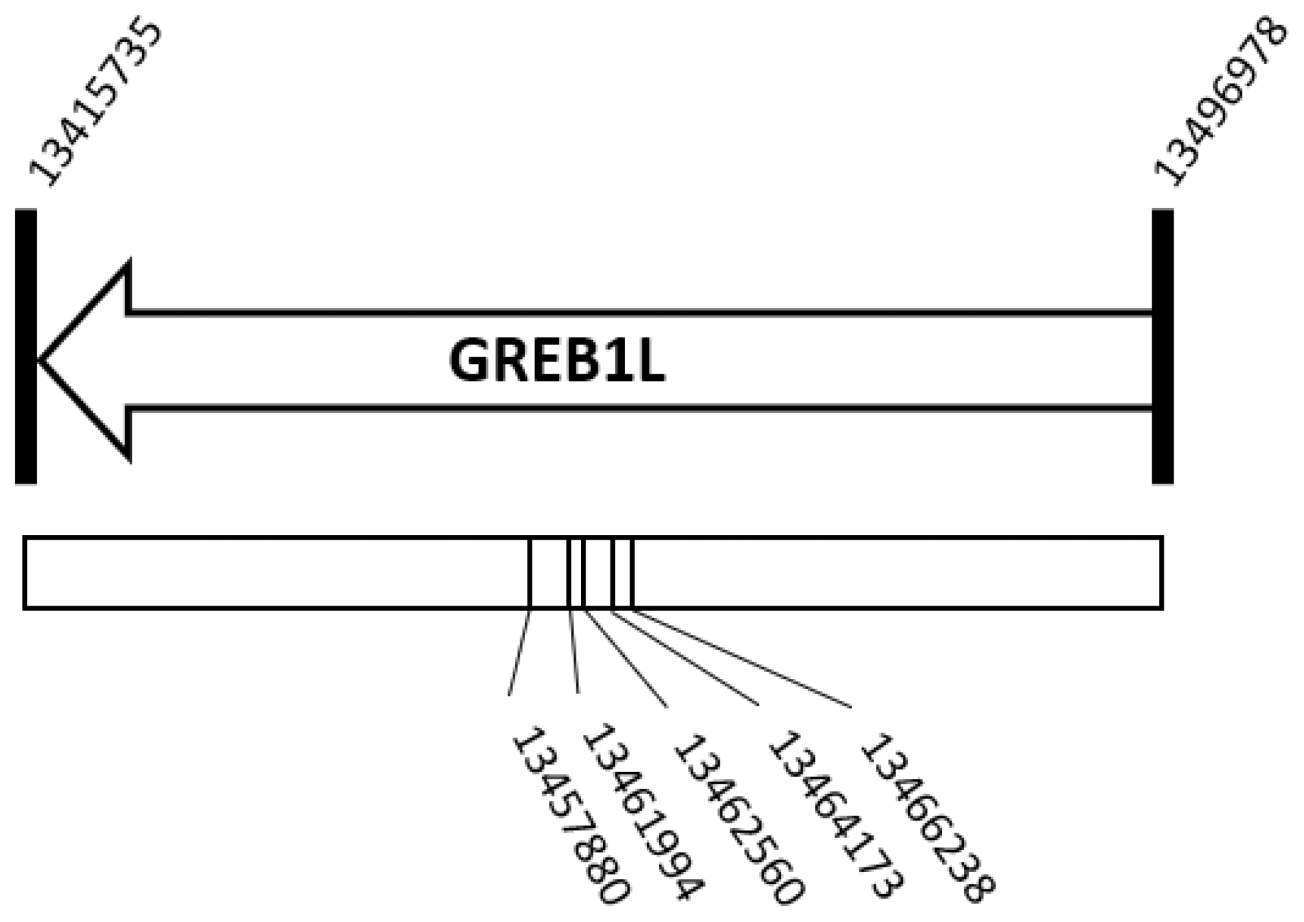
Diagram of relative SNP positions in the GREB1L region on chromosome 28 of the Chinook salmon genome, Otsh_v2.0 (GCF_018296145.1), used for genotyping analysis (Christensen et al., 2018).

To genotypically assign a run‐type, we extracted DNA from fin clips using the DNeasy® Blood and Tissue extraction kit (Qiagen, Valencia, CA). We genotyped fish at a specific region of GREB1L previously shown to be the most highly associated with Chinook salmon run timing in Central Valley populations (Thompson et al., 2020) by selecting five Single Nucleotide Polymorphisms (SNPs) across this region that had been identified as strongly associated with run timing in previous analyses (Koch & Narum, 2020; Thompson et al., 2020). As the causative variant/s remain unknown and the linkage between a given marker and the causative variant/s may not be complete, genotyping five SNPs rather than one or two provides greater confidence in run‐time calls. Input design sequences (Table S1) were cross‐checked against a multi‐population dataset utilized by Thompson et al. (2019) to screen out non‐target polymorphisms that could potentially disrupt the assay efficacy. We developed the SNPs into Fluidigm SNP‐type assays. Individuals were genotyped at the five SNPs using the Fluidigm EP1 platform (Figure 2). From those markers, we were able to make assignments to either homozygous early, homozygous late, or heterozygous genotypes. Individuals were only allowed to have one missing or discordant SNP genotype, and all other successful genotypes were required to agree. Otherwise, calls were deemed ambiguous and reported as “not called.” Those samples were not included in the final analyses.

### 2.4 Statistical analysis

We calculated the mean return date for each run using the day of year converted to the ordinal date of detection in the system by each of the three methods: hook‐and‐line surveys, acoustic tagging, and carcass surveys. To test if there was a significant difference in mean detection date for each of the three genotypes within each survey method, we used a Kruskal–Wallis test due to the unequal variance among sampling dates (Hollander & Wolfe, 1973). After determining whether the differences between the distribution of detection dates for the genotypes were significant, we then ran a Dunn test (Dunn, 1964) of significance using a Bonferroni correction to see which genotype mean and median detection dates specifically were significantly different from each other within each method, with a full pairwise comparison: homozygous early versus heterozygous, heterozygous versus homozygous late, and homozygous late versus homozygous early.

## 3 RESULTS

**FIGURE 3:**
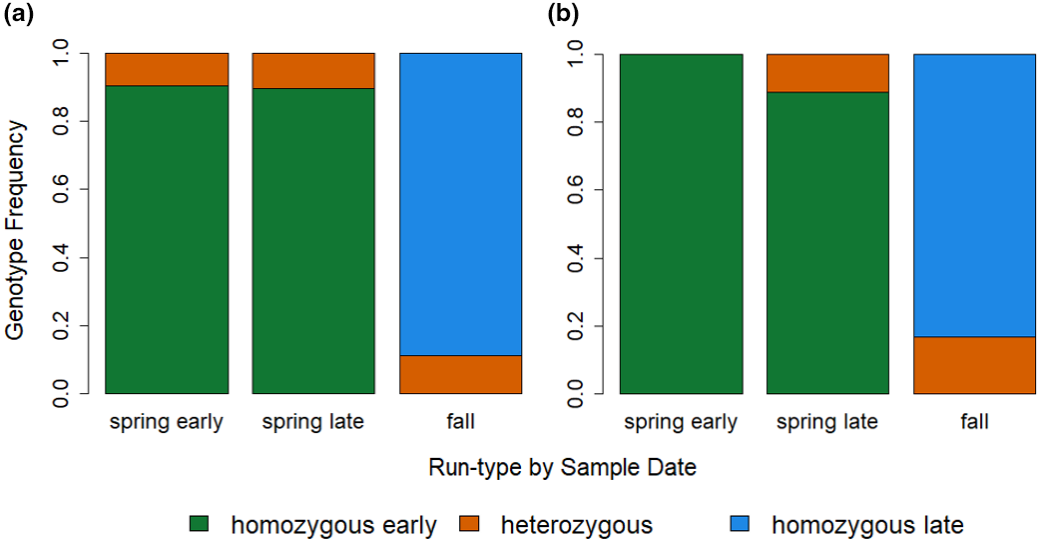
Stacked bar graphs of GREB1L genotyped proportions of individuals sorted into phenotypes classified by when they entered the system as spring early (before July 15th), spring late (after July 15th but before October 1st), or fall (after October 1st) using (a) fish surveyed when they first arrived in the system below DPD (spring early = 74, spring late = 39, fall = 9), and (b) fish in A that were acoustically tagged by the date they passed DPD (spring early = 9, spring late = 27, fall = 5).

**FIGURE 4:**
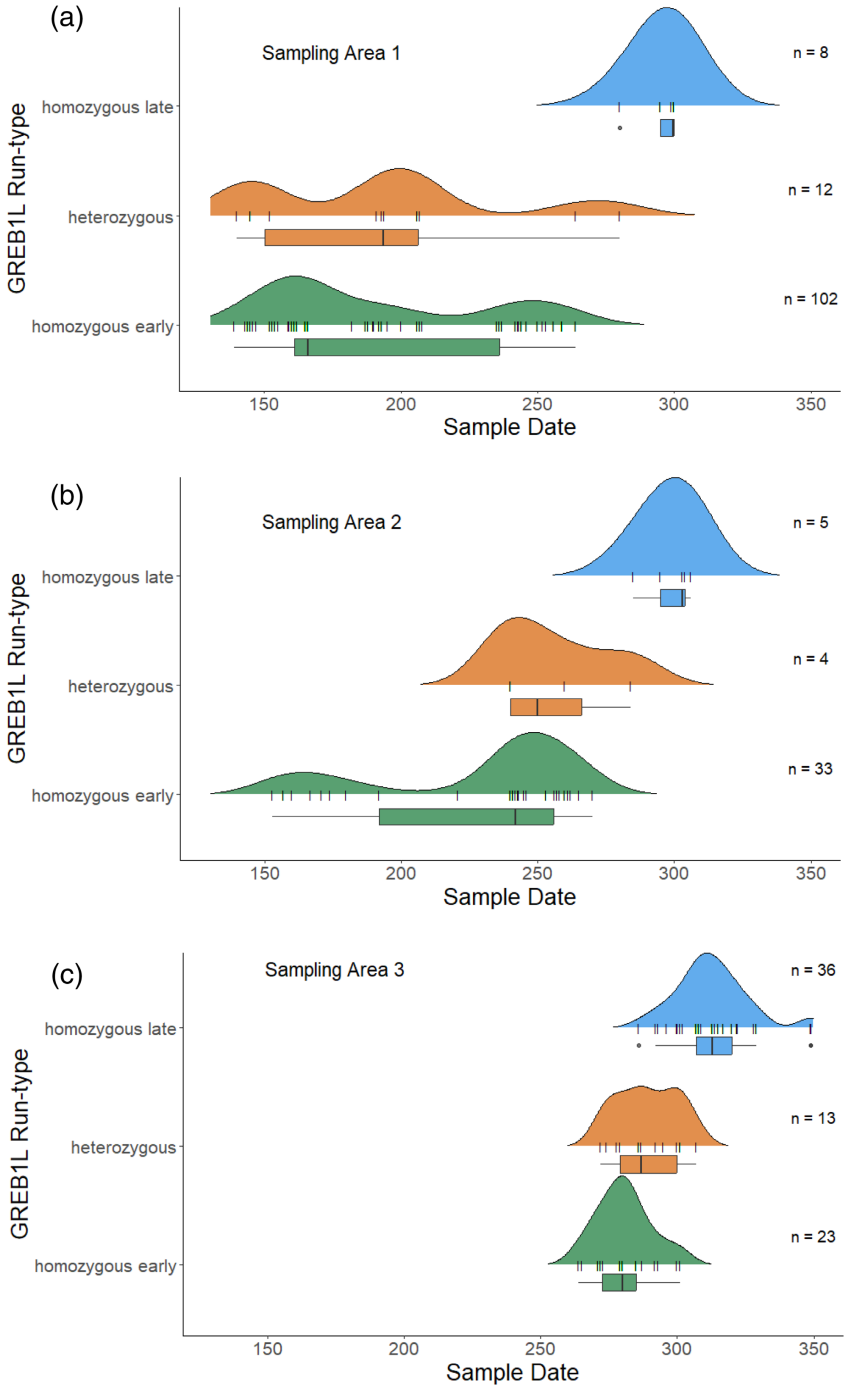
Genotypic assignments plotted against date of entry into the Yuba River system colored by GREB1L genotype and median return date using (a) fish surveyed as they entered the Yuba River below DPD, (b) acoustically tagged fish in Panel (a) that passed DPD, and (c) fish that were detected in carcass surveys, post‐spawn. The sample date is in ordinal days, with the equivalent calendar days as follows: 150 = May 30th and day 350 = December 16th. Each notch on the ridge portion of the plot represents one individual fish sampled. Bar plots below each ridge graph show the median, 25th percentile, and 75th percentile.

Within the Yuba River, genetic assignments show there are genetically spring‐run (GREB1L homozygous early), fall‐run (GREB1L homozygous late), and GREB1L heterozygous individuals in the system. In total, we found 125 homozygous early, 25 heterozygous, and 44 homozygous late individuals. All individuals used in analyses from this point on were required to be concordant at four out of the five SNPs per genetic assignment, with 169 of the 194 samples successfully genotyping concordant at all five SNPs. When compared with survey data, we found that genetic versus date‐assigned run types were not in perfect agreement. We found homozygous early individuals in both the spring early and spring late migrant phenotypic categories, while homozygous late individuals show up in the fall phenotypic category (Figure 3). Interestingly, heterozygous individuals appear below DPD at the same time as homozygous early individuals and were categorized as spring early and spring late based on sample date (Figure 3a); however, all heterozygous fish with acoustic tags crossed DPD later in the season. This caused them to be categorized as spring‐late and fall based on sample date (Figure 3b). We found that this was likely because although homozygous early and heterozygous individuals arrive at the dam at the same time (as early as May 25th, Figure 4a), they cross the dam at different time periods, with homozygous early fish crossing the dam earliest (as early as June 30th). We did not see the heterozygous individuals crossing the dam until later (at the earliest by August 28th, Figure 4b). For the post‐spawning carcass surveys, we saw a similar, albeit less protracted pattern, with homozygous early being detected at earlier dates, homozygous late being detected at later dates, and heterozygous individuals being detected at intermediate times (Figure 4c).

Our results clearly show that homozygous early individuals cross the dam earlier while homozygous late individuals cross the dam later in the season, with the mean return date being statistically significantly different (*p* = 0.0004). The same pattern was statistically significant across all sampling methods, with homozygous late mean return dates being later than homozygous early (hook‐and‐line survey: *p* = 0.0067, carcass survey: *p* = 5.58 e − 11). Across all sampling methods, heterozygous mean migration dates were not significantly different from homozygous early, despite slight differences in the mean migration date (Table 2). The statistical differences between median and mean return dates did not differ, so we report only the mean here.

**TABLE 2:** Statistical results for Kruskal–Wallis comparisons and the Dunn test of detection date for each of the three collection methods, comparing within each method for each of the three genotype classifications, where * indicates a significant value.

Survey type	Genotype	Mean return date	Median return date	Kruskal– Wallis *χ* ^2^	Kruskal–Wallis *p*	Comparison	Dunn test *p*
Hook‐and‐line Survey	Homozygous early	198.41	166	10.2395	0.01*	Homozygous early/heterozygous	0.3039
	Heterozygous	183.13	193.5			Heterozygous/homozygous late	0.0022*
	Homozygous late	298.33	299.5			Homozygous late/homozygous early	0.0067*
Acoustic Tagging	Homozygous early	227.03	242	13.458	0.001*	Homozygous early/heterozygous	0.7779
	Heterozygous	256.00	250			Heterozygous/homozygous late	0.0670
	Homozygous late	298.60	303			Homozygous late/homozygous early	0.0004*
Carcass Survey	Homozygous early	280.57	280	46.933	<0.0*	Homozygous early/heterozygous	0.2883
	Heterozygous	289.08	287			Heterozygous/homozygous late	0.0001*
	Homozygous late	312.75	313			Homozygous late/homozygous early	5.58e‐11*

## 4 DISCUSSION

This study provides direct evidence of spring‐run Chinook salmon in the Yuba River and further validation that the GREB1L run timing genotypes are correlated with early or late river sample date. Our data show that individuals entering the system early in the season are genetically homozygous for the early migrating alleles or heterozygous, while individuals that enter the system late are homozygous for the late migrating alleles. From the acoustic tagging data collected, it appears that heterozygous individuals are passing the dam at a slightly intermediate time point, even though they first appear in the system at the same time as homozygous early‐running fish. We recognize that our sample numbers for heterozygotes are lower than one would prefer (Figure 4, Table S2), and additional acoustic tagging would assist in further elucidating the strength of these relationships; however, given the extremely threatened nature of these fish and their very low population sizes, we think the information provided by these samples is incredibly valuable. Additionally, the fact that we did not find more heterozygotes in this system also points to the maintenance of these distinct life histories and genotypes, despite homogenizing anthropogenic influences. Although we could not eliminate fish from the Feather Hatchery completely from our analysis as only 25% of fall‐run fish in the CCV have their adipose fin clipped, isotopic evidence shows that Yuba origin spring and fall‐run fish are returning to the river, as opposed to Feather River Hatchery‐origin fish (Willmes et al., 2024). This is encouraging, as it indicates that there is hope for an independent, genetically distinct Yuba River spring‐run population.

We show there is clearly a pattern of homozygous early genotypes entering the system early through all survey methods. In addition, we see a clear and significant difference in spawning time between homozygous early and homozygous late that maintains their temporal segregation in spawning time despite the elimination of spatial separation. Although it is plausible that the carcasses were not surveyed until after fish had entered the system, we are certain that surveys were carried out weekly and decomposition rates in this system are fast enough for us to be confident that these fish were sampled relatively soon after they had spawned and not in the system for many additional days beyond the date of spawning. It is also important to note that although we did find many comparisons to be significant, a lack of symmetry in the data and unequal variances, particularly in the sample distribution of the homozygous early fish, can cause unreliable results from Kruskal–Wallis test comparisons. We still do find the pattern of early and late return dates to be quite striking, even given this caveat. We also recognize that these fish were not sampled as they first entered freshwater, and the correlation between GREB1L genotypes and migration timing tends to deteriorate as fish are sampled higher in the watershed and later during their migration (Waples et al., 2022). It is unlikely that early migrants oversummered for a period downstream of the Yuba, as they historically oversummer at as high an altitude as possible to take advantage of appropriately cool water pools, and no other habitat below the Yuba River has sufficiently cool water for adult Chinook salmon to hold over summer. Given that Chinook salmon returning to the Yuba River historically had access to higher spawning grounds before the construction of Englebright dam and that the lower reaches likely provided important oversummering habitat, we find it entirely plausible and very likely that entry into the Yuba River system could be an appropriate proxy for early and late returning entry into freshwater.

Our validation of the relationship between GREB1L genotypes and migration phenotypes in the Central Valley is noteworthy because it means GREB1L can be used to detect, monitor, and quantify the presence of different runs in the Central Valley. The advent of SHERLOCK, which allows especially fast, economical, and field deployable genotyping of the GREB1L locus, makes this possibility even more feasible and has the potential to revolutionize our ability to understand and monitor Chinook salmon life history diversity throughout the Central Valley (Baerwald et al., 2020). In addition, the results found in this study and the combination of tagging and carcass surveys could be used to provide spring‐run spawner abundance estimates each year, which is critical information for managing spring‐run separately from fall‐run fish.

Our study also shows that although the dam has eliminated spatial separation between the runs, creating some overlap between the presence of spring and fall returning individuals in the system, it does appear that time of entry in the system can also be used as a proxy to determine run type in the Yuba River. Our research shows that despite anthropogenic influence and very limited to no historical access to spring‐run spawning habitat due to dam construction, there are still both spring and fall returning populations that are genetically distinct and temporally separated from each other in the Yuba River. This temporal separation is likely only possible due to cold water pools above the DPD and below the Englebright Dam that allow for spring‐run fish to survive over the summer and spawn (Pasternack et al., 2010). It is encouraging that the Yuba River has maintained a spring‐run population, indicating that important diversity needed to maintain federally listed populations still exists within this altered landscape. It is also possible that in the years since these samples were collected, the amount of spring‐run has further decreased, although we expect the genetic conclusions to remain the same. Unfortunately, populations have been excluded from large areas of historic oversummering habitat, and the remaining habitats are predicted to disappear with a warming climate, leaving only the north Yuba River as potential habitat for spring‐running fish (Cordoleani et al., 2021). To ensure the persistence of spring‐run fish, it will be necessary to continue monitoring efforts to maintain and manage cold water access for these populations.

Discovering that genetically distinct early migrants exist within the Yuba River provides evidence that the system may be able to recover if appropriate conservation efforts and management actions are taken. There is currently an agreement among state, federal, and local officials to reopen large portions of habitat for Yuba River fish. This planned restoration includes the testing and creation of a comprehensive reintroduction plan to reintroduce CCV spring‐run Chinook salmon into the upper Yuba River habitats as well as habitat restoration design to allow more natural passage around Daguerre Point Dam (California State Government, O. of the G, 2023). This is an important step toward spring‐run Chinook salmon recovery; however, given the impending threats posed by climate change, further actions may be required to ensure that spring‐run populations recover and persist. Research has shown that intraspecific diversity within spring‐run Chinook salmon is critical for responding to changing climatic conditions, particularly increases in river and ocean temperatures, helping populations to maintain the biocomplexity necessary for resilience and persistence (Cordoleani et al., 2021). More research is needed to fully understand how diversity in migration timing, particularly within the spring‐run, contributes to an overall portfolio effect, but this will likely be curtailed by a lack of available habitat (Sturrock et al., 2019). Because spring‐run Chinook salmon rely on cool water to hold over during the summer months, this makes them more susceptible to future threats and continued anthropogenic change such as climate change and water diversion (Meyers et al., 1998; National Research Council, 2004; Quinn et al., 2016). It will therefore be important to ensure that any management actions in the Yuba River promote both the genetic and phenotypic diversity, as well as the hydrological conditions needed to support that diversity.

The Central Valley is a complex and highly altered system with many historical and contemporary threats to life history diversity in fishes (Fisher, 2016; Williams, 2006). However, our work shows that altered ecosystems can still sustain genetic and life history diversity. Life history diversity in salmon has been especially important to maintain species resiliency and persistence and will continue to be of high importance as we experience more development and more extreme climate regimes (Beechie et al., 2006; Bourret et al., 2016; Pearson et al., 2014). It is often assumed that systems where subpopulations are extirpated or contain introgressed individuals are lacking or have lost life history diversity and biocomplexity. Without a full understanding of the variation in genotypes and phenotypes in degraded systems, it is all but impossible to manage them to maintain this diversity. This study highlights the importance of identifying, monitoring, and protecting diversity, even in highly altered environments. In order to ensure the persistence and resilience of populations in the face of climate change, it will be necessary to protect the little diversity that is left before it is lost forever.

## CONFLICT OF INTEREST STATEMENT

No conflicts of interest to declare.

## Supporting information


Table S1.–S2.

## Data Availability

The raw data underlying the main results of this study are archived in the Dryad Digital Repository https://doi.org/10.5061/dryad.fj6q57436.
