# Supplementary material for: Remnant salmon life history diversity rediscovered in a highly compressed habitat
Source: Evol Appl. 2024 Jul 2;17(7):e13741. doi: 10.1111/eva.13741 (PMC11217596; doi:10.1111/eva.13741)
Supplement: Supplementary file 1 — Table S1.–S2. [file EVA-17-e13741-s001.docx]

Supplementary Table 1: Single Nucleotide Polymorphisms used in Fluidigm type assays from the GREB1L region with their name, genomic positions, original publication, and sequence.

| SNPtype Fluidigm Name | Otsh_v1.0_NC_scaffold | Otsh_v1.0_NW_scaffold | Otsh_v2.0 | Original publication identifying SNP | Sequence used as input for Fluidigm assay design |
| --- | --- | --- | --- | --- | --- |
| GREB1l_pos2194538 | NC_037124.1:12273002 | chr28_NW_020128528.1\|:2194538 | NC_056456.1:13457880 | Thompson et al. (2020) | GATAAGGGGATAAGGGAGGTCATGCAAATTCCATACCATCCAGGTCAGACAGTGCTAGAACTTTAACCGGAACGCTGCATGAGTTTAGGGAACATTCTCTTTAGTA[T/C]CAGACTGAACATCCAAATCTTCCTTCACTTCTAGATACACGCTTTAAGGGCCCTCTAGGCAGCTAACTCTGCATCCACAGTAATATAACCCATTCTAGGAGACATTCTTATAACACTGGCCTAGACTACAAATCACTCTTAACATAACCCTGTAGCTGTGTCCATGATCACAGGGTCACTATCAA |
| GREB1l_pos2198644 | NC_037124.1:12277108 | chr28_NW_020128528.1\|:2198644 | NC_056456.1:13461994 | Koch & Narum (2020) | TTTGTCTTCCATTGATATTTGACCTCATGTGGATGTGCCAATGACAACATTATTATTCTCACTCTTAAATCCAACATTAGGGAGACTTAAAACAACCTCAAAAGAGCTACACAATATATTCACGATAACACCATATGTCGYTTGTYTCCTTCACCTGCAACCTTCTATTCAACAGTCCATTCTTAGAAAAATGACAAGCCYGAGTAAGCCAGTCGGTGAGCCATTCATAACAATCTTAACATTACTTT[T/A]CAAAAATATTGGATTCGGAATATGGATTCATAACATAATTATGTTATCCTGGATCATTCAAGAGAAATGAACAGACGGATGAAACATTAAGTCAGAGGATGTTGATCATGACCATATTGTTTAACTGTAATTCTTTCATTTTCATCTTTGCATAGCCAGGCAAGCCGTGTGACTGACTGACTGCCTTAGTCTTCAGTTCATTACAGCAGATCTAGTCAACAGTTGGTTTAATCTGTCCGTATAACTCTTCTCACCTCCT |
| GREB1l_pos2199210 | NC_037124.1:12277674 | chr28_NW_020128528.1\|:2199210 | NC_056456.1:13462560 | Koch & Narum (2020) | CTCCACACCACTCATTCATCATACACACATCGCGCATTCTATGCTGAACSTGGCGGTTCGTGTCCATTGCATTATTATACGACACAGCGTCTGTCTSTCTGWATGGACTCTRTAGGCTCCCGGGGGTAGTCCATTTGAAACAGTTGGAGTAAAGAATGAAAGAAAGAGATGACTTGTKCCCTAAGAGGAGACGAGCATTACAGTTAGTAAACATTACAGTTTCC[T/A]GTCTGAGGTAAATCAACATATGACCACTCGAAAACTCCCCAAATAAGCTCATTTGGTACAGACCAGCACTAGCAGCAAGTTCAACCTGGGAAGAGGAGTCTCACGGKGTGATTAATCTCCCCCAGCTCCCAGCAGTAGCTCCCTCCCTCCCYGACTTTGACACAGCAGCCACGTTTAAATAGACCCGTTTGAAATGAAGATAATGAGTAAACCCAGCGGTTTCTTTGGCCTCAAAMGAGCCCTGTGTGGGAAAACAAAAGAGCCT |
| GREB1l_pos2200828 | NC_037124.1:12279292 | chr28_NW_020128528.1\|:2200828 | NC_056456.1:13464173 | Koch & Narum (2020) | TAAGGGTTGTGGGTGGTGGGGTGGATTAGCCAGTGGGGACTATAAAGGGGAGTGAACTAGGGTTTAAGGCCTGTTGTGACAGAGGAGCTGGGGAAGGGCTGATGGGGGGGCKGGGGGGAGGCGGACAAAAGGAGCATTTGGGCAGATGAAGAAGTCATCATCATTAAGCCACTGGAAGTTTACTGTCCAGTTATAAAAGTCATTTCAAAATTAGGRGGTTAGGGGGTGCGTGTGAAAGG[G/A]GAGAAGGGCTCAGAGTGCCTGAGAAGGCCTGGGGGYGGGGCAGATGAGAGCTGTGGCCTGTGGTTGTGAGGGACTCTGTGGGACTGGGGGGCCAATTCATTAGGGGCACAGCCCAGCCTTTGTGTTTGCACCAGGTTGATTGGAGTGCTGACCTTGCCTTGCCTCCCAGCCTTCCCTGCACACTCTGCTCTGGCCCAGTGGAGGATGAGTATAAGGGCAAGGCATTTAACCTTCAACTAAATCCCAGCCTCAACCACAGCAGACAAAGG |
| GREB1l_pos2202893 | NC_037124.1:12281357 | chr28_NW_020128528.1\|:2202893 | NC_056456.1:13466238 | Thompson et al. (2020) | ATTTACCTCCCTGCCCCAGACAATTCTTGAATCACATGGCTGCTGCATTTCATAATGAAAAACAAGGCCA[A/T]ATCAGGAAGTTCAGCCCTCTTTAAATGTGGAAAAMAAAATACAKAGAACATTTTCACTTAGTGTTGTTCTTTTTAAATTTAATTTGAGGCCTGGAGGACAAACTCAATCAATGTGCGGAATTACTGATAATTGACCATGCTCGCTGAGAAGGCCRAATAAAATTGAAGCCCTGAKTGAACCCGCTCTGCATTTTACAACACTGC |

Supplementary Table 2: Number of samples in each genotype category organized by each type of survey. Note that samples from individuals in the that were in the acoustic tagging survey were also included in the spawning survey, since that is where they were first detected.

| Survey Type | Homozygous early | Heterozygous | Homozygous Late |
| --- | --- | --- | --- |
| Spawner Survey | 102 | 12 | 8 |
| Acoustic Tagging | 33 | 4 | 5 |
| Carcass Survey | 23 | 13 | 36 |
| Total | 92 | 21 | 41 |
